# ISSLS prize in basic science 2021: a novel inducible system to regulate transgene expression of TIMP1

**DOI:** 10.1007/s00586-021-06728-0

**Published:** 2021-02-01

**Authors:** Yingchao Han, Zhihua Ouyang, Richard A. Wawrose, Stephen R. Chen, Maximiliane Hallbaum, Qing Dong, Emily Dando, Ying Tang, Bing Wang, Joon Y. Lee, Jeremy D. Shaw, James D. Kang, Gwendolyn A. Sowa, Nam N. Vo

**Affiliations:** 1 Department of Spine Surgery, School of Medicine, Renji Hospital, Shanghai Jiao Tong University, Shanghai, China; 2 Department of Spine Surgery, School of Medicine, Shanghai East Hospital, Tongji University, Shanghai, China; 3 Ferguson Laboratory for Orthopaedic and Spine Research, Department of Orthopaedic Surgery, University of Pittsburgh, Pittsburgh, PA USA; 4 Department of Spine Surgery, The First Affiliated Hospital of Nanhua University, Hengyang, China; 5 Molecular Therapeutics Laboratory, Department of Orthopaedic Surgery, University of Pittsburgh, Pittsburgh, PA USA; 6 Department of Physical Medicine and Rehabilitation, University of Pittsburgh School of Medicine, Pittsburgh, PA USA; 7 Department of Orthopaedics, School of Medicine, Brigham and Women’s Hospital, Harvard University, Boston, MA USA

**Keywords:** Intervertebral disc degeneration, Gene therapy, TIMP1, Regulated transgene expression, NFκb

## Abstract

**Purpose:**

Inflammatory and oxidative stress upregulates matrix metalloproteinase (MMP) activity, leading to intervertebral disc degeneration (IDD). Gene therapy using human tissue inhibitor of metalloproteinase 1 (hTIMP1) has effectively treated IDD in animal models. However, persistent unregulated transgene expression may have negative side effects. We developed a recombinant adeno-associated viral (AAV) gene vector, AAV-NFκB-hTIMP1, that only expresses the hTIMP1 transgene under conditions of stress.

**Methods:**

Rabbit disc cells were transfected or transduced with AAV-CMV-hTIMP1, which constitutively expresses hTIMP1, or AAV-NFκB-hTIMP1. Disc cells were selectively treated with IL-1β. NFκB activation was verified by nuclear translocation. hTIMP1 mRNA and protein expression were measured by RT-PCR and ELISA, respectively. MMP activity was measured by following cleavage of a fluorogenic substrate.

**Results:**

IL-1β stimulation activated NFκB demonstrating that IL-1β was a surrogate for inflammatory stress. Stimulating AAV-NFκB-hTIMP1 cells with IL-1β increased hTIMP1 expression compared to unstimulated cells. AAV-CMV-hTIMP1 cells demonstrated high levels of hTIMP1 expression regardless of IL-1β stimulation. hTIMP1 expression was comparable between IL-1β stimulated AAV-NFκB-hTIMP1 cells and AAV-CMV-hTIMP1 cells. MMP activity was decreased in AAV-NFκB-hTIMP1 cells compared to baseline levels or cells exposed to IL-1β.

**Conclusion:**

AAV-NFκB-hTIMP1 is a novel inducible transgene delivery system. NFκB regulatory elements ensure that hTIMP1 expression occurs only with inflammation, which is central to IDD development. Unlike previous inducible systems, the AAV-NFκB-hTIMP1 construct is dependent on endogenous factors, which minimizes potential side effects caused by constitutive transgene overexpression. It also prevents the unnecessary production of transgene products in cells that do not require therapy.

## Introduction

An estimated 84% of adults experience low back pain during their lifetime [1]. Of the many causes for low back pain, intervertebral disc degeneration (IDD) is a common contributor [2]. IDD results from an imbalance between catabolism and anabolism of the disc extracellular matrix (ECM) [3]. Loss of the ECM proteoglycan leads to dehydration and fibrosis of the nucleus pulposus (NP), which commonly results in formation of fissures in the annulus fibrosis (AF) [4]. These disc fissures are not only responsible for the abnormal biomechanical changes seen in IDD, but are also associated with neoinnervation, which has been implicated in pain [5].

The pathways that regulate intervertebral disc matrix homeostasis provide potential targets for gene therapy. For example, Le Maitre et al. [6] delivered an interleukin-1 (IL-1) receptor antagonist through a gene therapy approach and noted inhibition of matrix degradation. Recently, our lab introduced human tissue inhibitor of metalloproteinase 1 (hTIMP1) and bone morphogenetic protein (BMP) into the NP of rabbits and demonstrated delayed disc degeneration in a rabbit annulotomy model of IDD [7]. hTIMP1 has demonstrated promise as a therapeutic factor to ameliorate IDD. hTIMP1 inhibits the activities of most of the known matrix metalloproteinases (MMPs), the catabolic enzymes involved in degrading the ECM and also facilitates collagen and aggrecan expression in the disc [8–11].

Despite these encouraging results, the treatment of nonlethal diseases, such as IDD, with gene therapy presents unique safety concerns. Specifically, previous gene therapy studies have used cytomegalovirus (CMV) promoters to drive transgene expression, resulting in constitutively elevated protein expression that may have negative metabolic consequences. For example, Wallach et al. [12] noted severe neurological changes and significant histological changes in rabbits after intradural injection of gene constructs that resulted in elevated levels of TGF-β1. To avoid such unfavourable sequelae, controlled inducible transgene expression systems have been developed. We previously demonstrated tetracycline-inducible and RheoSwitch-inducible expression systems that successfully modulated transgene expression in NP cells [13, 14]. Unfortunately, these systems would be difficult to use clinically since gene expression requires an exogenous activator ligand.

Another concern with gene therapy is that viral vectors, specifically adenovirus (AV) vectors, may trigger severe immune responses that can have devastating consequences, as demonstrated by the death of a patient treated for an enzymatic deficiency with an AV vector [15]. Additionally, the immune response degrades the adenoviral episome, which leads to a discontinuation of therapeutic protein production [16, 17]. To mitigate the immune response, adeno-associated virus (AAV) vectors have become more popular. AAV vectors differ from AV vectors in that they require a helper virus to be expressed, which limits expression of AAV gene products and the resulting cellular mediated immune response [18].

The aim of this study was to establish a gene expression system that produces hTIMP1 protein only under conditions of stress. We achieved this using a novel transgene regulation system, AAV-NFκB-hTIMP1. The system was built on an AAV backbone with hTIMP1 expression regulated by a mini-promoter containing NFκB elements, which is turned on by inflammatory and oxidative stress and turned off upon diminishment of cellular stress [19].

## Materials and methods

### Overview of the study

**Fig. 1:**
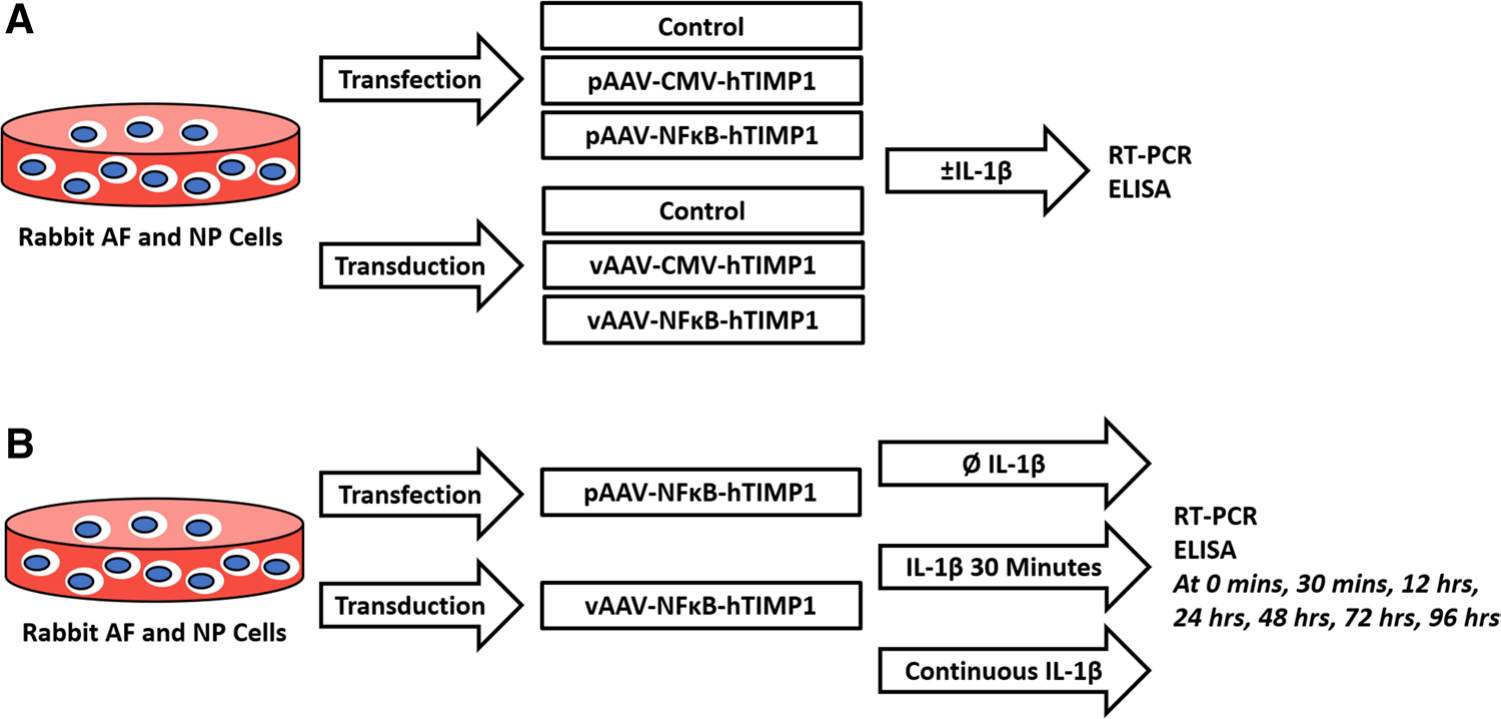
**a** Identification of an inducible transgene expression system in response to IL-1β. Rabbit disc cells were transfected or transduced with AAV-CMV-hTIMP1 or AAV-NFκB-hTIMP1. Cells were selectively exposed to IL-1β. RT-PCR and ELISA measured mRNA and protein expression 24 h after IL-1β exposure. **b** Demonstration of the time course of the hTIMP1 transgene expression. Rabbit disc cells were transfected or transduced with AAV-NFκB-hTIMP1. Cells were selectively exposed to IL-1β for either 30 min or continuously. RT-PCR and ELISA were used to measure mRNA and protein expression at different time points after stimulation

Rabbit disc cells were transfected with AAV-CMV-hTIMP1 or AAV-NFκB-hTIMP1 plasmid DNA (pAAV-CMV-hTIMP1 or pAAV-NFκB-hTIMP1), or transduced with AAV-CMV-hTIMP1 or AAV-NFκB-hTIMP1 viruses (vAAV-CMV-hTIMP1 or vAAV-NFκB-hTIMP1). Disc cells were treated with IL-1β to induce inflammatory stress to activate the NFκB signaling pathway. Reverse transcription polymerase chain reaction (RT-PCR) and enzyme-linked immunosorbent assay (ELISA) were used to determine the levels of expression of hTIMP1 messenger RNA (mRNA) and protein, respectively. To test the temporal expression of hTIMP1 in response to stress, pAAV-NFκB-hTIMP1 transfected cells or vAAV-NFκB-hTIMP1 transduced cells were either transiently stimulated with IL-1β for 30 min—after which IL-1β was removed—or continuously stimulated with IL-1β for 96 h. hTIMP1 expression was measured in culture media and cell lysate at 0, 0.5, 12, 24, 48, 72, and 96 h after IL-1β treatment. The study design is summarized below (Fig. 1**)**.

### Construction of plasmids and production of viruses

**Fig. 2:**
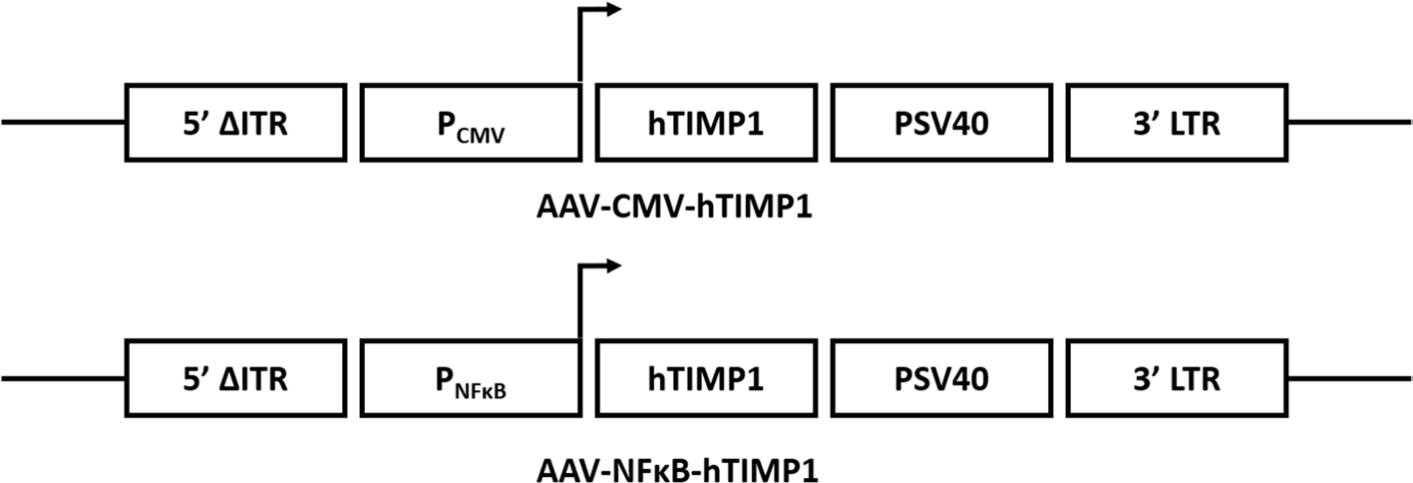
Recombinant adeno-associated viral (AAV) vectors. Schematic illustration of two AAV vectors containing hTIMP1 gene (624 bp). Vectors were controlled by either the human CMV immediate-early promoter/enhancer (PCMV) or the human NFκB regulatory elements (PNFκB) and then, followed by the simian virus 40 polyadenylation signal (PSV40) in the self-complementary AAV vector. ∆ITR, truncated inverted terminal repeat; LTR, long terminal repeat

The full length of functional hTIMP1 gene (0.62 kb) was derived from our previously published construct, AAV-CMV-hTIMP1 [7]. hTIMP1 was cloned into a regulated AAV vector driven by the NFκB promoter, AAV-NFκB-hTIMP1. Both AAV-NFκB-hTIMP1 and AAV-CMV-hTIMP1 gene constructs were utilized, with the latter serving as a control for constitutive hTIMP1 expression (Fig. 2). Plasmids were purified through CsCl_2_ gradient ultracentrifugation according to previously published protocols [20].

Recombinant AAV viral vector stocks were produced according to the three-plasmid co-transfection method as described by Xiao et al. [21]. The viral stocks were obtained by CsCl_2_ gradient centrifugation. The titers of viral preparations used in the study were between 1 × 10^12^ and 10^13^ particles/mL.

### Disc cell culture

AF and NP cells were isolated from New Zealand white rabbits immediately after sacrifice and cultured in F-12, 10% fetal bovine serum, 1% penicillin–streptomycin at 37 °C, and 5% CO_2_ until 90% confluence, as previously described [22]. Passage 1 cells were seeded on 12-well plates and expanded in monolayer for three days in normoxic conditions for subsequent transfection and transduction as described below.

### Transfection of pAAV-NFκB-hTIMP1 and pAAV-CMV-hTIMP1

pAAV-NFκB-hTIMP1 and pAAV-CMV-hTIMP1 were transfected into rabbit AF (rAF) and rabbit NP (rNP) cells using Xfect Transfection Reagent (Clontech Laboratories, Inc., Palo Alto, CA) according to the manufacturer’s instructions. Briefly, rAF and rNP cells were seeded into 12-well plates at 25 k cells/well with a complete cell culture medium and cultured to 50% confluence. Xfect Polymer (0.75 μl/well) was mixed with pAAV-CMV-hTIMP1 or pAAV-NFκB-hTIMP1 (2.5 μg/well) and incubated at room temperature for 10 min to allow nanoparticle complexes to form. Transfection complexes were added to cells and incubated in a 37 °C/CO_2_ incubator.

### Transduction of vAAV-NFκB-hTIMP1 and vAAV-CMV-hTIMP1

rAF and rNP cells were seeded into 12-well plates at 25 k cells/well with a complete cell culture medium and cultured to 50% confluence. 10–15 µl virus stock of vAAV-CMV-hTIMP1 or vAAV-NFκB-hTIMP1 was added to each well. Plates were placed in a 37 °C/CO_2_ incubator.

### NFκB activation

Nuclear translocation of NFκB was detected using a fluorescent microscope at time points 0, 0.5, 12, 24, 48, and 72 h after IL-1β treatment. NFκB was visualized by immunofluorescent labelling using an anti-p65 antibody (Santa Cruz Biotechnology, Santa Cruz, CA). Nuclear translocation of NFκB was quantified using a customized MATLAB (The MathWorks, Inc, Natick, MA) program to analyze the images. The experiment was conducted in triplicate.

### Inducible transgene expression

To assess whether transgene expression was inducible by IL-1β, cells were divided into six groups: cells alone without IL-1β, cells alone with IL-1β, pAAV-CMV-hTIMP1 without IL-1β, pAAV-CMV-hTIMP1 with IL-1β, pAAV-NFκB-hTIMP1 without IL-1β, and pAAV-NFκB-hTIMP1 with IL-1β. If indicated, 10 ng/mL of IL-1β was added at 24 h post-transfection. Supernatants and cells were collected for RT-PCR, ELISA, and MMP activity assay after treatment with IL-1β for 24 h. This experiment was repeated for cells that were transduced with vAAV-CMV-hTIMP1 or vAAV-NFκB-hTIMP1.

### Time course of transgene expression

To assess the time course of the hTIMP1 transgene expression, cells transfected with pAAV-NFκB-hTIMP1 were divided into three groups: cells not treated with IL-1β, cells treated with IL-1β transiently for 30 min, and cells treated continuously with IL-1β for 96 h. If indicated, 10 ng/mL of IL-1β was added at 24 h post-transfection. Supernatant and cells were collected for RT-PCR and ELISA at 0, 0.5, 12, 24, 48, 72, and 96 h after IL-1β treatment. This experiment was repeated for cells that were transduced with vAAV-NFκB-hTIMP1.

### hTIMP1 mRNA expression

For mRNA isolation, cells in PBS were detached from the plate by mechanical disruption. RLT lysis buffer containing 1% β-mercaptoethanol was added to the cells. The resultant solution was passed through a QIAshredder (Qiagen, Valencia, CA), and mRNA was isolated using an RNA extraction kit (Qiagen, Valencia, CA) with a DNase I step to remove genomic material. hTIMP1 mRNA expression was measured by RT-PCR using primer pair 5′-TGGCTTCTGGCATCCTGTTGTTG-3′ and 5′-CGCTGGTATAAGGTGGTCTGGTTG-3′. Relative changes in mRNA were quantified using the ΔΔCt method using glyceraldehyde 3-phosphate dehydrogenase (GAPDH) to normalize and compare between groups [23].

### hTIMP1 protein expression

hTIMP1 protein expression in cell culture supernatants were measured by hTIMP1 ELISA Duoset (R&D Systems, Minneapolis, MN) according to the manufacturer’s instructions. Briefly, 2 × 10^6^ cells were seeded into 60 mm dishes. After all cells attached, culture medium was replaced with fresh medium for 24 h. Cell culture supernatants were harvested after centrifugation to remove cell debris and diluted to be within the range of the standard curve.

### MMP enzymatic activity

MMP activity was assayed using 520 MMP Fret Substrate XI (AnaSpec, Fremont, CA). Cell-conditioned media (CM) samples were divided into two sets: 1 mM APMA was added to one set of APMA-activated CM samples and PBS was added to the second set of non-activated control CM samples. Immediately after adding APMA/PBS, 520 MMP Fret Substrate XI solution was applied to all samples and fluorescence (Ex/Em = 355/460 nm) was measured on a BioTek microplate reader every 5 min over 1 h. MMP activities were determined as the differences between sample fluorescence measurements and those of corresponding vehicle controls. A linear fit model was used to determine the rate of the cleavage reaction. Cleavage rates were compared both among samples and between APMA-activated and non-activated sample treatments.

### Statistical analysis

Student’s *t*-test with significance set to *p* < 0.05 was used to identify differences in mRNA expression, protein expression, and MMP enzymatic activity between control cells, cells with the AAV-CMV-hTIMP1 plasmid, and cells with the AAV-NFκB-hTIMP1 plasmid. Student’s *t*-test with significance set to *p* < 0.05 was used to identify differences in mRNA expression and protein expression in cells transfected with pAAV-NFκB-hTIMP1 or cells transduced with vAAV-NFκB-hTIMP1 that were untreated, treated with IL-1β for 30 min, or treated with IL-1β continuously for 96 h.

## Results

### NFκB activation in response to inflammatory stimulus in disc cells

**Fig. 3:**
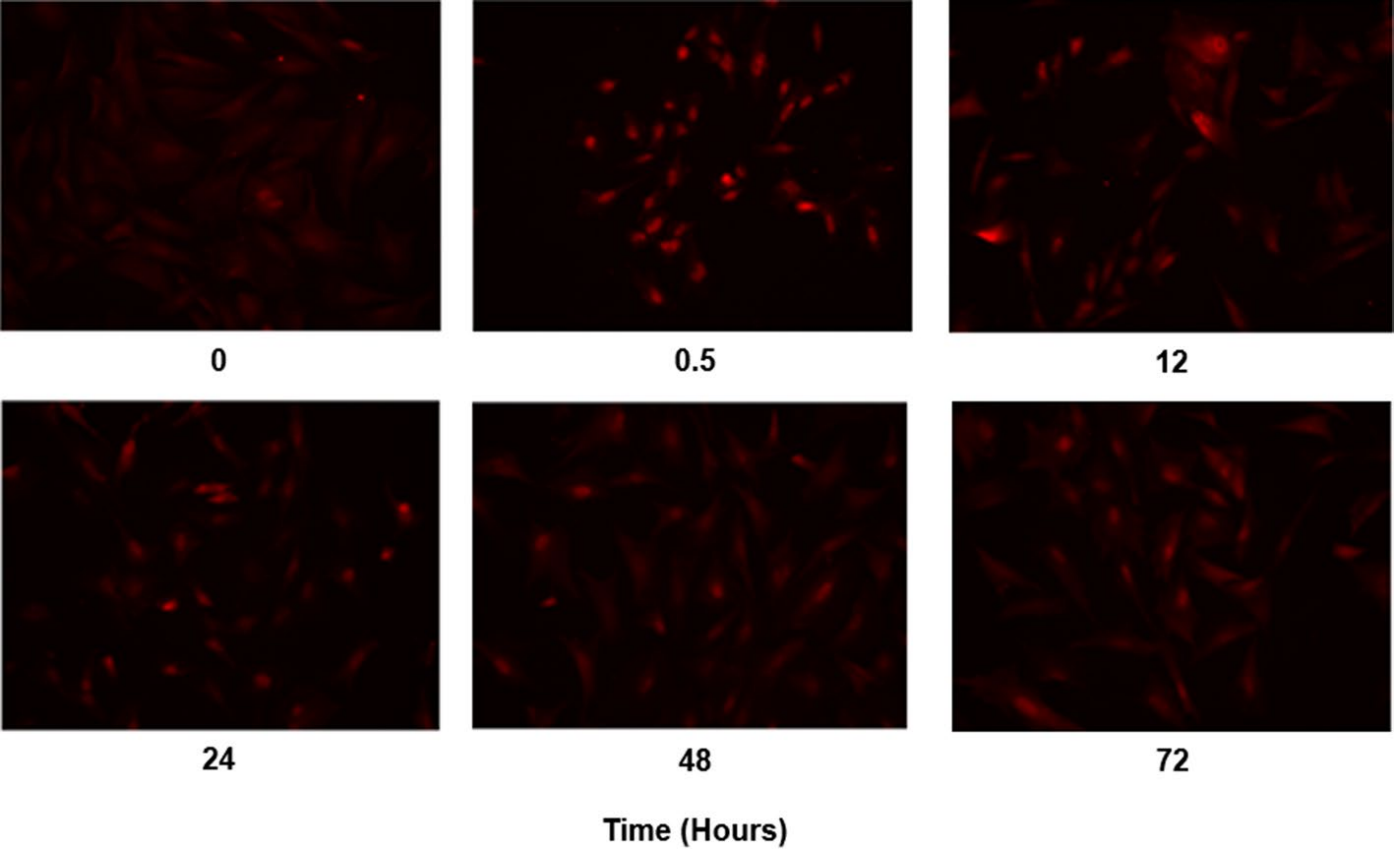
NFκB activation by IL-1β in NP cells. Immunofluorescence of p65, a subunit of NFκB, was measured at 0, 0.5, 12, 24, 48, and 72 h after cells were treated with 10 ng/mL of IL-1β. Cells exhibited complete translocation of p65 from the cytoplasm to the nuclei 30 min after IL-1β treatment. p65 nuclear localization gradually decreased at the later time points

The transgene was designed to be regulated by the NFκB signalling pathway, which responds to inflammatory stress. To confirm that IL-1β was an appropriate surrogate for inflammatory stress, NP cells were exposed to IL-1β and nuclear translocation of NFκB from the cytoplasm was assessed. In IL-1β treated cells, NFκB was activated—indicated by increased NFκB nuclear translocation—30 min after IL-1β treatment (Fig. 3**)**. NFκB activation remained, albeit at modestly decreased levels, from 12 to 72 h after IL-1β treatment, which is consistent with the dynamics of NFκB activation reported previously [24, 25]. These findings demonstrated that the NFκB signalling pathway can be robustly activated by the pro-inflammatory cytokine IL-1β in disc cells, which justifies the use of IL-1β to investigate the expression of the hTIMP1 transgene from AAV-NFκB-hTIMP1.

### Inflammatory stress upregulates hTIMP1 gene and protein expression in disc cells harbouring the AAV- NFκB-hTIMP1 DNA construct

**Fig. 4:**
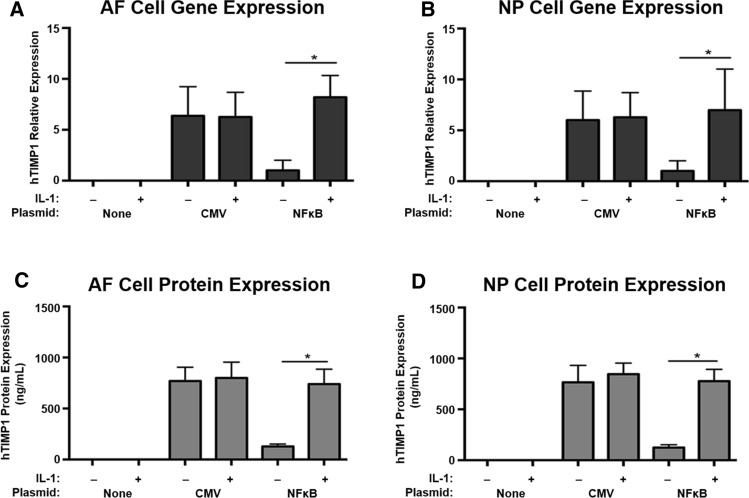
hTIMP1 mRNA and protein expression in disc cells transfected with plasmid vectors as measured by RT-PCR and ELISA. In cells transfected with NFκB promotor vectors, hTIMP1 expression was induced by IL-1β with little expression at baseline. In contrast, there was noted to be high hTIMP1 expression in cells with CMV promotor vectors regardless of stimulus. * = a value significantly different from that of other groups (*p* < 0.05)

To evaluate regulated expression of hTIMP1 in our new transgene system, rAF and rNP cells were transfected with either pAAV-NFκB-hTIMP1 or pAAV-CMV-hTIMP1. pAAV-CMV-hTIMP1 served as a positive control for high production of hTIMP1 from the constitutively expressed CMV promoter. As expected, pAAV-CMV-hTIMP1 transfected disc cells highly expressed hTIMP1 mRNA and protein in an unregulated manner, regardless of the absence or presence of IL-1β (Fig. 4). hTIMP1 mRNA expression as measured by RT-PCR from pAAV-NFκB-hTIMP1 transfected disc cells was negligible in the absence of a stressful stimulus, i.e. IL-1β, in both rAF (Fig. 4a) and rNP cells (Fig. 4b). With IL-1β treatment, hTIMP1 mRNA expression increased five- to six-fold in pAAV-NFκB-hTIMP1 transfected disc cells to a level comparable to the constitutive expression seen in pAAV-CMV-hTIMP1 transfected disc cells. hTIMP1 protein levels as determined by ELISA mirrored those of hTIMP1 mRNA (Fig. 4c, d). Negative control cells, which were not transfected, did not express any hTIMP1 mRNA or protein. These results suggest that hTIMP1 expression from pAAV-NFκB-hTIMP1 is upregulated only under conditions of stress.

**Fig. 5:**
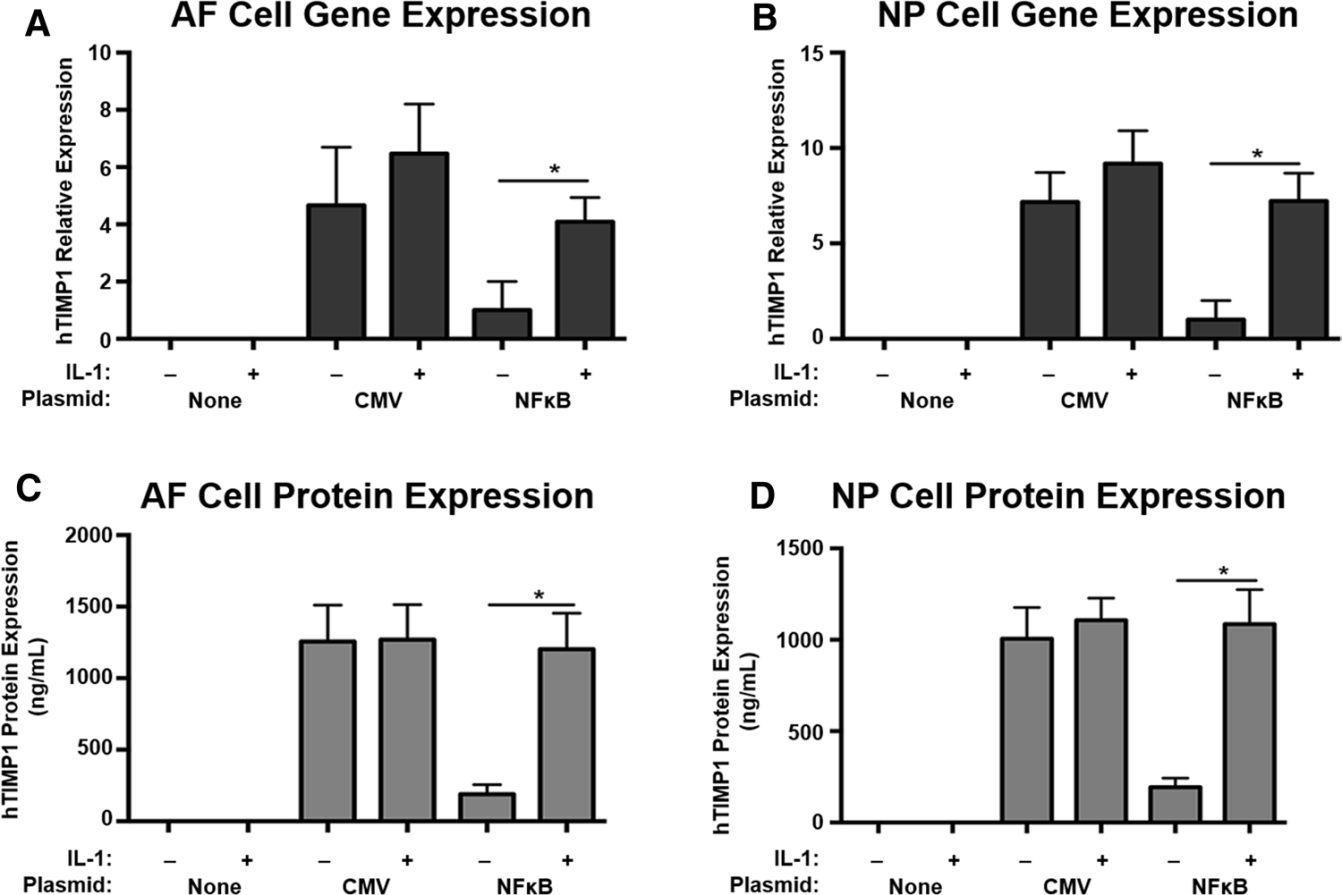
hTIMP1 mRNA and protein expression in disc cells transduced with viral vectors as measured by RT-PCR and ELISA. Cells transduced with viral vectors displayed similar pattern to cells transfected with plasmid vectors, confirming that expression was secondary to the NFκB-mediated promoter’s selective response to IL-1β. * = a value significantly different from that of other groups (*p* < 0.05)

Regulated expression of hTIMP1 in our new transgene system was also confirmed in rAF and rNP cells transduced with vAAV-NFκB-hTIMP1 or vAAV-CMV-hTIMP1. hTIMP1 gene and protein expressions in vAAV-NFκB-hTIMP1 transduced disc cells were similar to those observed in pAAV-NFκB-hTIMP1 transfected disc cells, i.e. negligible hTIMP1 expression at baseline and high expression of hTIMP1 with IL-1β stimulation. The negative controls had no expression of hTIMP1 and the positive controls under the constitutive promoter transduced with vAAV-CMV-hTIMP1 had high expression regardless of IL-1β stimulation (Fig. 5).

### Upregulated hTIMP1 gene expression from AAV-NFκB-hTIMP1 suppressed MMP enzymatic activity in disc cells

hTIMP1 has been used as a therapeutic to minimize pathologic proteolytic degradation of the ECM by MMPs. Hence, it is important to evaluate if hTIMP1 expression from the AAV-NFκB-hTIMP1 construct also regulates MMP activity in disc cells. Non-transfected disc cells exhibited a low level of baseline MMP enzymatic activity, which was upregulated three-fold in the presence of IL-1β. These results demonstrate that under condition of stress without the presence of MMP inhibitors such as hTIMP1, there is a significant increase in MMP activity in disc cells, as expected.

**Fig. 6:**
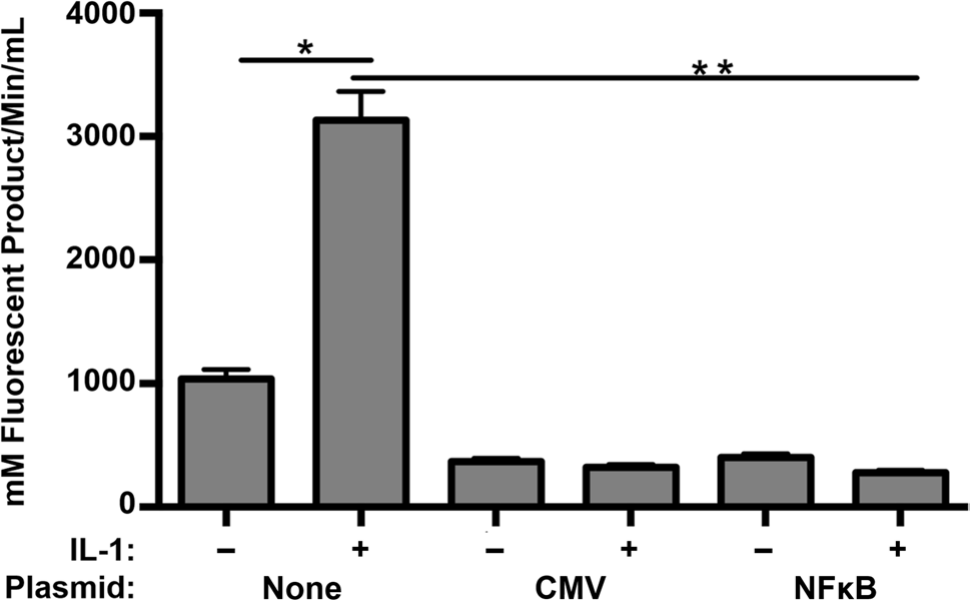
MMP enzymatic activities in response to IL-1β in control cells and those transfected with either pAAV-CMV-hTIMP1 or pAAV-NFκB-hTIMP1. The level of MMP activity was decreased in the cells transfected with pAAV-NFκB-hTIMP1 as compared to baseline levels or cells exposed to IL-1β

Upregulated hTIMP1 expression in the presence of IL-1β in pAAV-NFκB-hTIMP1 transfected disc cells dramatically blunted the observed increase in MMP enzymatic activity (Fig. 6). Similar suppression of MMP activity was seen in pAAV-CMV-hTIMP1 transfected disc cells in which hTIMP1 was constitutively expressed. Disc cells transfected with pAAV-NFκB-hTIMP1 in the absence of IL-1β also exhibited minimal MMP activity, confirming that the transfection itself did not result in increased MMP activity.

### Downregulation of hTIMP1 expression following IL-1β withdrawal in disc cells harbouring the AAV-NFκB-hTIMP1 DNA construct

**Fig. 7:**
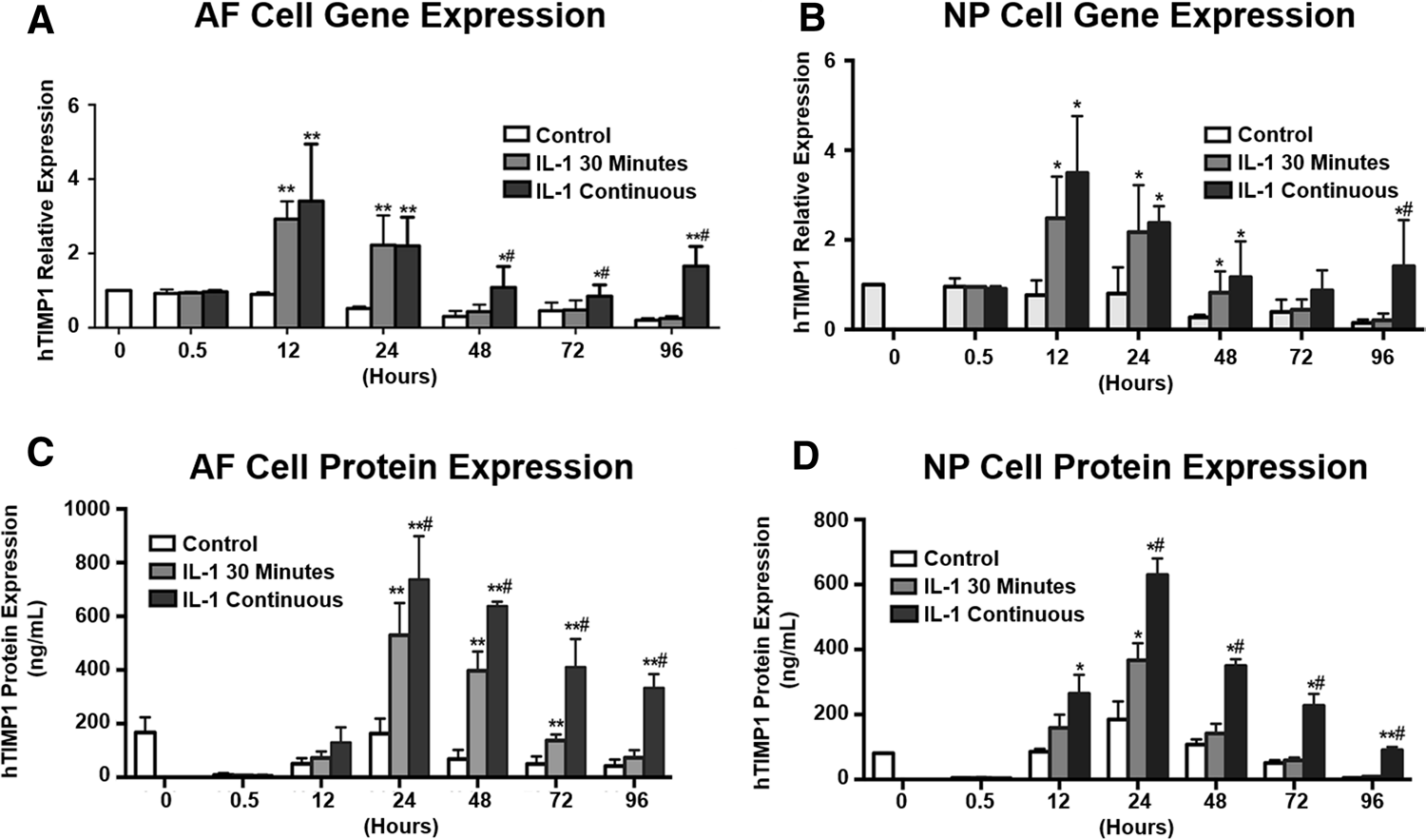
Decreased hTIMP1 expression in AF and NP cells transfected with pAAV-NFκB-hTIMP1 following IL-1β withdrawal. The mRNA and protein levels of hTIMP1 were significantly increased after IL-1β stimulation. The mRNA and protein levels of hTIMP1 returned to near baseline levels with IL-1β withdrawal

The AAV-NFκB-hTIMP1 construct was designed to express hTIMP1 transgene only under conditions of stress. hTIMP1 indeed was expressed in the presence of IL-1β stimulation and not in the absence of IL-1β (Figs. 4, 5). However, the question remained as to whether IL-1β-induced expression of hTIMP1 would diminish in AAV-NFκB-hTIMP1 containing disc cells once the stressful stimulus was removed. This idea was tested by exposing pAAV-NFκB-hTIMP1 transfected disc cells to IL-1β for 30 min, then removing IL-1β from the culture media and quantifying hTIMP1 expression at different time points following IL-1β withdrawal. As a positive control, AAV-NFκB-hTIMP1 transfected disc cells were treated with IL-1β continuously for 96 h. As a negative control, untreated AAV-NFκB-hTIMP1 transfected disc cells were included. rAF and rNP cells transfected with pAAV-NFκB-hTIMP1 and exposed to 30 min of IL-1β demonstrated peaked hTIMP1 mRNA and protein expression within 12–24 h compared to the negative control group. More importantly, the levels of hTIMP1 mRNA and protein progressively decreased in the transiently stimulated cells after 24 h and reached a minimum at 96 h after IL-1 withdrawal (Fig. 7).

In cells continuously stimulated with IL-1β for 96 h, hTIMP1 mRNA and protein expression were generally increased at all time points within the 24- to 96-h period when compared to the transiently stimulated group. The negative control group demonstrated consistently low expression of hTIMP1 in both AF and NP cells (Fig. 7).

**Fig. 8:**
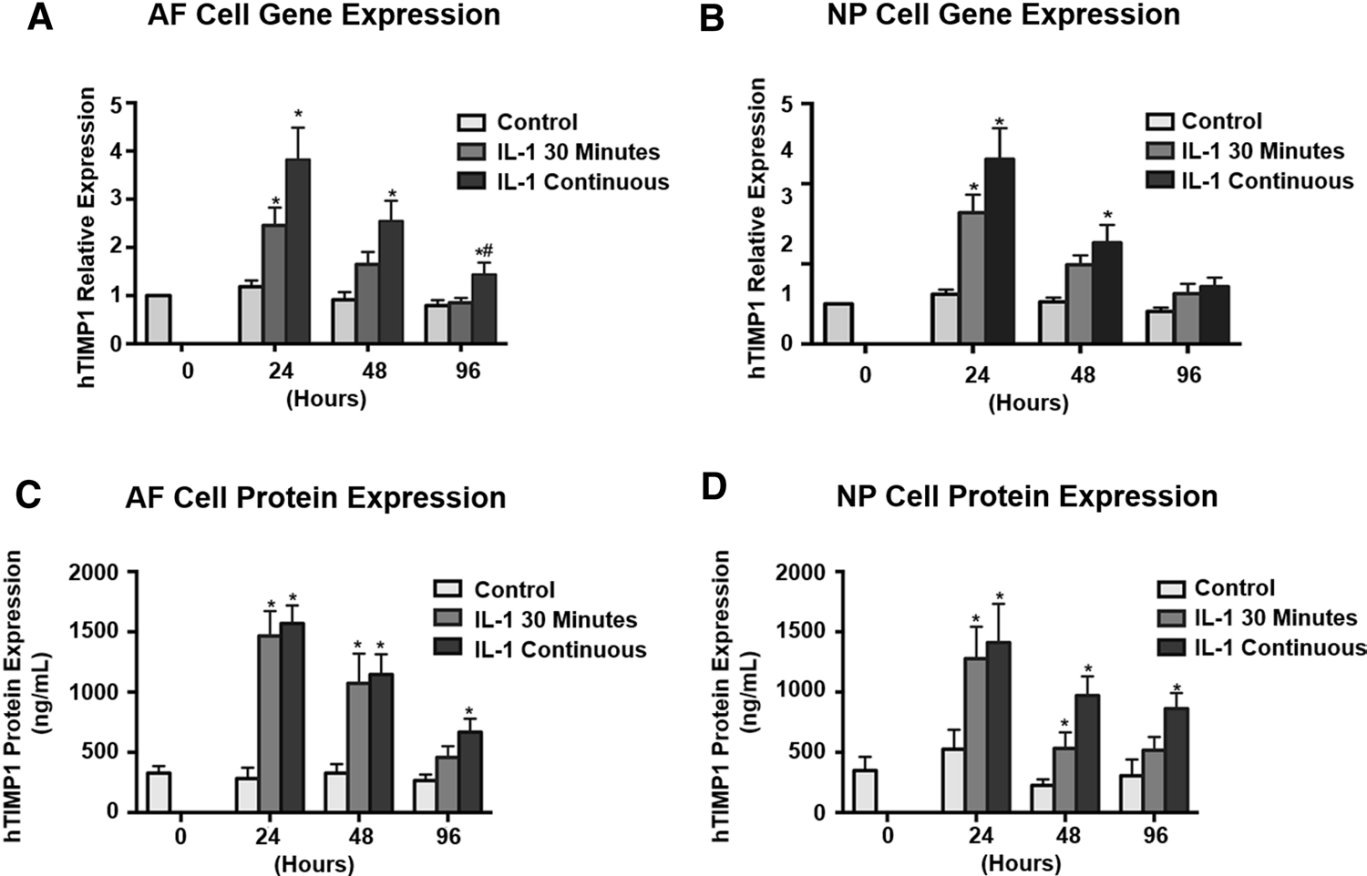
hTIMP1 expression in AF and NP cells transduced with vAAV-NFκB-hTIMP1 following IL-1β withdrawal. mRNA and protein levels of hTIMP1 were significantly increased after IL-1β stimulation. The mRNA and protein levels of hTIMP1 returned to near baseline levels with IL-1β withdrawal

It is possible that the reduced hTIMP1 expression after IL-1β withdrawal was not only due to decreased NFκB activation but also due to transient transfection of the plasmid, i.e. loss of plasmid from the cells over time. To address the possible loss of plasmids from cells due to transient transfection, we performed the same experiments except with transduction instead of transfection. Downregulation of hTIMP1 mRNA and protein expression following IL-1β withdrawal was confirmed in rAF and rNP cells transduced with vAAV-NFκB-hTIMP1 (Fig. 8). These results were almost identical results to those in cells transfected with pAAV-NFκB-hTIMP1, which implies that downregulation of hTIMP1 expression is due to withdrawal of inflammatory stress and not transient transfection of cells.

## Discussion

Past IDD gene therapy studies used unregulated expression vectors with constitutively expressed transgenes. While these studies showed promise, there was concern regarding unregulated, constitutive expression of transgene proteins, particularly for growth factors where overexpression can cause undesirable side effects [12]. In addition, previous attempts to control transgene expression relied on an exogenously added bio-signal (e.g. tetracycline) to stimulate or inhibit expression. This study demonstrates the proof of concept of an intrinsic inducible transgene expression system to treat IDD by regulated production of hTIMP1 in disc cells, responsive to the internal environment in the disc, potentially improving the overall safety and effectiveness of intradiscal gene therapy. The NFκB promoter allows controlled hTIMP1 expression, i.e. hTIMP1 gene is turned on under conditions of stress and off in the absence of cellular stress, avoiding untoward side effects of constitutive expression and associated metabolic stress on disc cells. Compared to the traditional CMV-driven gene therapy strategy, this regulated system prevents bioenergetic overtaxing from chronic overexpression of the transgene and adverse effects of accumulated transgene protein products.

As demonstrated in Figs. 4 and 5, disc cells transfected or transduced with AAV-CMV-hTIMP1 with the constitutive CMV promoter resulted in unregulated constitutive expression of hTIMP1, while disc cells harbouring AAV-NFκB-hTIMP1 exhibited hTIMP1 expression only in the presence of IL-1β, demonstrating that the system is inducible. Furthermore, upregulation of transgene expression by IL-1β highlights the potential applicability of our regulated gene therapy strategy to treat inflammatory-related conditions, such as IDD, induced only under conditions of inflammation. Of note, previous measurements of IL-1β in degenerated human disc cells range from 34.9 to 135.5 pg/mL, which is well below the 10 ng/mL amount used in this experiment [24]. However, IL-1 levels as low as 100 pg/mL, similar to the levels seen in vivo in degenerated discs, are reported to activate inflammatory pathways, such as NFκB [25, 26]. Additionally, other pro-inflammatory cytokines that activate NFκB, such as TNF-α, are present in degenerated disc cells. Furthermore, NFκB activation is well documented in degenerative discs in vivo [27–29]. Thus, although confirmatory in vivo studies are needed, it is highly likely that expression of the hTIMP1 transgene in the AAV-NFκB-hTIMP1 vector system would be induced in degenerated discs in vivo.

Perhaps more importantly, our inducible system turns off when the source of stress is removed, providing an additional safety feature to prevent constitutive expression. After stimulation of disc cells by IL-1β both hTIMP1 mRNA expression and protein production prominently increase. In the transiently stimulated group, cells treated with IL-1β for only 30 min, hTIMP1 mRNA and protein levels started to decline 12 and 24 h after the first dose of IL-1β, respectively. In the continuously stimulated group, hTIMP1 levels were higher than that of the transiently stimulated group throughout 96 h. It should be mentioned that hTIMP1 expression did decrease in the continuously stimulated group. This decrease could be due to either degradation of the plasmid or the oscillatory nature of NFκB activation by IL-1β, whereby NFκB activates its own repressor resulting in activity that oscilates with time [30, 31]. While this characteristic may cause difficulty maintaining a constant amount of gene product, there is significantly more gene product being produced in those cells maintained in a pro-inflammatory state.

It should be noted that disc cells transfected or transduced with the NFκB-hTIMP1 plasmid exhibited some basal hTIMP1 expression compared to non-transfected or non-transduced cells. Such basal hTIMP1 expression accounted for decreased MMP activity in disc cells containing pAAV-NFκB-hTIMP1 when compared to non-transfected disc cells. It is likely that the basal hTIMP1 expression observed in disc cells transduced or transfected with AAV-NFκB-hTIMP1 was due to the cellular stress associated with the transduction or transfection procedure.

This study is limited to testing the novel regulated system in in vitro cell cultures. Future i*n vivo* studies are required to demonstrate the clinical use of this inducible transgene system in restoration of disc matrix and amelioration of IDD. In vivo studies will also present new challenges, specifically how gene therapy will be delivered to disc cells. A minimally invasive procedure such as an injection would be preferable. However, there are complications associated with injections including volume constraints, damage to the AF, and cell leakage [32]. Despite these limitations, this study established a novel inducible system to regulate transgene expression of hTIMP1. The novel inducible system ensures that hTIMP1 expression is high only under conditions of stress, such as inflammation, that activates NFκB signalling. This inducible system facilitates the creation of new approaches towards effective and safe gene therapy to IDD and opens the door for future studies analyzing molecular therapeutics of disc degeneration. This study serves as a proof of concept. It is possible that genes other than hTIMP1 could be integrated into the inducible system extending its application beyond the treatment of IDD to other inflammatory disease states.
